# S217. SELF-DISTURBANCES AND DIAGNOSTIC STABILITY IN FIRST EPISODE PSYCHOSIS: A SEVEN YEAR FOLLOW-UP STUDY

**DOI:** 10.1093/schbul/sby018.1004

**Published:** 2018-04-01

**Authors:** Ingrid Svendsen, Merete Øie, Paul Møller, Barnaby Nelson, Ingrid Melle, Elisabeth Haug

**Affiliations:** 1 Innlandet Hospital Trust, Norway; 2 Vestre Viken Hospital Trust, Norway; 3 Orygen Youth Health Research Centre; 4 NORMENT Centre for Psychosis Research, University of Oslo

## Abstract

**Background:**

Self-disturbances are considered core features of schizophrenia spectrum disorders, and are present in the prodromal, the early psychotic and in the chronic phase. Self-disturbances are also present at first treatment in some patients with psychotic disorders outside of the schizophrenia spectrum. There is limited knowledge about the stability of self-disturbances over time. The aim is to explore the stability of self-disturbances in a seven year follow-up of first episode patients and to examine the association between self-disturbances at start of treatment and diagnostic changes at follow-up.

**Methods:**

Longitudinal study of 56 patients recruited at their first treatment for an affective or non-affective psychotic disorder. Self-disturbances were assessed by the Examination of Anomalous Self-Experience (EASE), while diagnostic categories, symptom severity, and functioning were assessed with standard clinical instruments. At baseline we registered life-time experiences of self-disturbances. At follow-up we focused on self-disturbances experienced the last two years

**Results:**

At follow-up 35 patients were diagnosed with schizophrenia or a schizoaffective disorder (schizophrenia) and 21 with a bipolar, psychotic disorder or delusional disorder (non-schizophrenia). The level of self-disturbances was significant lower at follow-up than at baseline in patients with schizophrenia. Patients with schizophrenia had significantly higher levels of self-disturbances both at baseline and at follow up than patients in the non-schizophrenia group, who showed stable low levels of self-disturbances. In the schizophrenia group the EASE domain “Cognition and stream of consciousness”, was the most stable. There were no changes into or out of the schizophrenia group. The four patients in the non-schizophrenia group with relatively high EASE total scores at baseline (≥ 15) did not convert to schizophrenia at follow-up, as hypothesized. No patients in the non-schizophrenia group who increased their EASE score from baseline to follow-up converted to the schizophrenia group.

**Discussion:**

EASE domain “Cognition and stream of consciousness”, have previously been described as some of the first self-disturbances appearing in the prodromal phase and are also found to be the most predictive of transition to full-threshold psychosis in an Ultra High Risk group. The results from the present study show that these phenomena are also the most stable over time. We did not find that patients outside the schizophrenia group, converted to schizophrenia, neither among those who had high level of self-disturbances at baseline nor those who had increased levels of self-disturbances at follow-up. The current study was conducted in rural areas with considerable distances to the specialized psychiatric health services, and consequently with long duration of untreated psychosis. The observed diagnostic stability is thus to be expected if symptomatic developments relevant for diagnosis take place early in the first episode, in this case before the first treatment contact.

